# Comparison of Polylactide-Based Active Films Containing Berberine and Quercetin as Systems for Maintaining the Quality and Safety of Blueberries

**DOI:** 10.3390/polym16111577

**Published:** 2024-06-02

**Authors:** Ewa Olewnik-Kruszkowska, Martina Ferri, Mariana C. Cardeira, Magdalena Gierszewska, Anna Rudawska

**Affiliations:** 1 Department of Physical Chemistry and Physicochemistry of Polymers, Faculty of Chemistry, Nicolaus Copernicus University in Toruń, Gagarin 7 Street, 87-100 Toruń, Poland; mgd@umk.pl; 2 Department of Civil, Chemical, Environmental and Materials Engineering (DICAM), University of Bologna, Via Terracini 28, 40131 Bologna, Italy; martina.ferri13@unibo.it; 3 National Interuniversity Consortium of Materials Science and Technology (INSTM), Via Giusti 9, 50121 Firenze, Italy; 4 Department of Chemistry, NOVA School of Science and Technology, FCT NOVA, Universidade NOVA de Lisboa, 2829-516 Caparica, Portugal; marianaccardeira@gmail.com; 5 Faculty of Mechanical Engineering, Lublin University of Technology, Nadbystrzycka 36 Street, 20-618 Lublin, Poland; a.rudawska@pollub.pl

**Keywords:** polylactide, thin films, active compounds, packaging materials

## Abstract

Polymeric thin films based on polylactide with an addition of poly(ethylene glycol) as a plasticizer and flavonoids in the form of quercetin and berberine were subjected to tests that were particularly relevant from the point of view of contact with food. A comparative analysis of the effect of individual flavonoids on the antioxidative properties of tested films and blueberry storage was carried out. The influence of active compounds on the water vapor permeability, as well as UV protection, of the obtained materials was investigated. Also, the specific migration of individual flavonoids from obtained materials to food simulants in the form of acetic acid and ethyl alcohol was determined. The crucial point of this study is the storage of blueberries. The obtained results indicate that the selection of packaging, containing individual active compounds, depends on the purpose and requirements that the packaging must meet for particular types of food.

## 1. Introduction

Packaging protects products from spoilage or physical damage. Packaging can also promote the product, enable its identification, and allow safe transport, storage, and use of a given product. Moreover, currently, modern packaging must perform additional, active functions, mainly to ensure and maintain high-quality food. Such packaging is called active packaging. In most cases, it contains substances that affect the atmosphere inside the package or interact with the packaged products. Flavonoids constitute one of the most promising groups of active compounds. Flavonoids are phytocompounds characterized by an extremely rich pro-health effect. Their name comes from the Latin word flavus, meaning “yellow”. They accumulate in leaves, flowers, and fruits (mainly in the outer tissue—the peel), giving them color, and often also taste and smell. However, the most essential features of flavonoids include antioxidative, anti-inflammatory, antibacterial, anti-cancer, and detoxifying properties [1]. Taking into account the above-mentioned advantages, some researchers described the application of flavonoids in forming active packaging [2,3,4,5,6].

The active compounds studied in this work are quercetin and berberine. Considering the most widely accessible food products, quercetin is mainly found in onions, broccoli, spinach, apples, blueberries, black currants, oranges, tea leaves, grapes (and therefore red wine), and honey. The popularity and properties of quercetin resulted in the publication of several papers in which it was introduced as an ingredient of active packaging. Tongdeesoontorn et al. [7] have analyzed cassava starch–carboxymethyl cellulose incorporating quercetin as active food packaging. It was established that the introduction of an antioxidant increased tensile strength but reduced elongation at break of the cassava starch–carboxymethyl cellulose films. This was caused by the intermolecular interactions between the polymer and the additive. The application of quercetin was studied in the work of Lopez-de-Dicastillo et al. [8]. Based on the obtained results, improving the antioxidant protection of packaged food by incorporating quercetin into ethylene-vinyl alcohol copolymer films was indicated. Moreover, quercetin, as an antioxidant and environmentally friendly indicator of aging time, due to a change in color, was analyzed by Masek et al. [9]. The aforementioned studies allowed us to formulate the conclusion that quercetin constitutes an effective natural antioxidant and an indicator that can be used in packaging materials.

Berberine is the second of the analyzed compounds found to influence the properties of the packaging and, consequently, fruit storage. Berberine can be obtained from barberry (*Berberis vulgaris* L.), goldenseal (*Hydrastis canadensis* L.), and Chinese goldsmith (*Coptis chinensis* L.). Its beneficial effect on the functioning of the body is widely known. Berberine supports liver function, has antibacterial, anti-inflammatory, and analgesic properties, has a relaxing effect on smooth muscles, gently lowers blood pressure, and lowers blood sugar and cholesterol levels. In addition, research is ongoing into the anti-cancer properties of berberine and its positive effect on neurodegenerative diseases. Berberine was also used in active packaging. Gelatin films incorporated with a berberine-based inclusion complex were studied in the work of Hu et al. [10]. It was established that films containing the mentioned additive displayed improved ultraviolet and visible light barrier properties. Moreover, the studied materials have very effective antimicrobial capacity. In the work of Ma et al. [5], active packaging consisting of gelatin and kappa-carrageenan films filled with berberine-based nanoparticles was investigated. The obtained results allowed confirmation that the formed packaging was characterized by significant antibacterial efficacy.

It should be noted that also in our previous publications [11,12], the structure and physicochemical properties of polymeric films based on polylactide with the addition of quercetin and berberine were studied. It was determined that the introduction of both—quercetin as well as berberine—grants antibacterial potential to the obtained packaging materials. The current study encompassed a comparison of packaging materials containing berberine and quercetin. For this reason, properties such as migration and resistance to water vapor permeation were investigated, and antioxidative and storage tests were performed. The mentioned properties are crucial in terms of packaging applications. In our opinion, the provided analyses can significantly broaden the knowledge concerning the improvement of food stability.

## 2. Materials and Methods

### 2.1. Material

Polylactide type 2002D, with an average molecular weight of 155,500 Da, was delivered by Nature Works^®^ (Minnetonka, MN, USA). Quercetin, berberine hydrochloride, and poly(ethylene glycol) were supplied by Sigma-Aldrich (Steinheim, Germany). Solvents such as chloroform, ethanol, acetone, methanol, and acetic acid were purchased from Avantor Performance Materials Poland S.A. (Gliwice, Poland). Commercially available solvents were used as received without further purification. 2,2-Diphenyl-1-picrylhydrazyl (DPPH) was purchased from Sigma Aldrich (Milano, Italy) and used as received.

### 2.2. Active Packaging Formation

**Figure 1:**
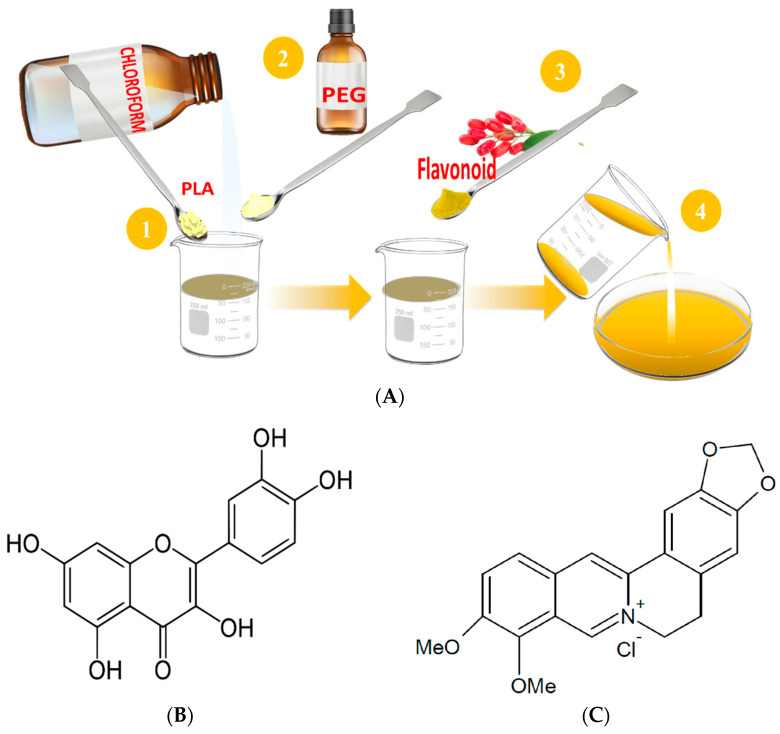
Scheme of the polymeric film formation (**A**), and the structures of (**B**) quercetin and (**C**) berberine.

Polylactide-based films were obtained using the procedure described in our previous works [11,12]. Firstly, polylactide pellets were dissolved in chloroform to form a 3% *w/v* solution. In the next stage, PEG1500 (5% *w*/*w*, PLA) was added and stirred until dissolution of the introduced plasticizer occurred. Finally, predetermined amounts of flavonoids (0.5%, 1%, or 2% *w*/*w*, PLA) were dissolved in a selected solvent (quercetin in acetone, berberine in methanol) and incorporated into the PLA-PEG mixture. The obtained solutions were stirred for 30 min and cast onto a glass plate. The scheme of polymeric film formation and the structures of used flavonoids are presented in Figure 1A–C.

The compositions of the obtained films are presented in Table 1.

### 2.3. Antioxidative Properties

The antioxidative properties of PG-B and PG-Q films were evaluated as radical scavenging activity (RSA) by employing the DPPH• free radical scavenging method [13,14]. A UV-visible spectrophotometer V-650, operating in the absorbance mode (Jasco, Tokyo, Japan) was used to record the absorption spectra of a DPPH• solution (0.2 mM in ethanol) over time, both with and without the addition of the films, in the form of small stripes (0.5 × 1.5 cm, thickness ≈ 80 µm). The times of measurement were 5, 10, 20, 30, 40, 60, 90, 120, 150, 210, 270, 330, 420 and 1440 min. Each spectrum was collected within the range of 700–200 nm (resolution of 0.5 nm). The antioxidative properties of each sample were calculated according to the following Equation (1):

(1) $$RSA\%=\left(1-\frac{A_{t_{x}}}{A_{t_{0}}}\right)\cdot 100$$

where $A_{t_{x}}$ is the maximum absorbance at each time of measurement after the introduction of the films, and $A_{t_{0}}$ is the maximum absorbance measured at *t*_0_, before the introduction of the films into the cuvette. Each result is presented as the mean value across three distinct samples.

### 2.4. Water Vapor Transmission Rate

The resistance of studied materials to water vapor transmission was determined according to the method described in our previous work [15]. The water vapor transmission rate (WVTR) was established by measuring changes in the calcium chloride weight every 24 h for a total of 7 days. It should be mentioned that samples were stored at 30 °C with a saturated NaCl solution (75% relative humidity). The values of WVTR were evaluated according to Equation (2):

(2) $$WVTR=\frac{Slope\;of\;the\;straight\;line\;}{Surface\;area\;of\;the\;film}\;\left[\frac{g}{m^{2}\cdot h}\right]$$

### 2.5. Migration Tests

Taking into account that the obtained materials can be applied as packaging materials for different food products, migration tests were performed. Based on the European Commission Regulation (EU) 2016/1416 [16], the appropriate conditions and contact with food simulants were analyzed. According to the regulation, the area-to-volume ratio was 6 dm^2^ of polymeric film per 1 kg of used simulant. The migration experiments were performed using two food simulants (3% *w*/*v* acetic acid solution (Food simulant B) and 10% *v*/*v* solutions of ethanol (Food simulant A)), in triplicate for 10 days at 40 °C. In the case of studied polymeric materials, the specific migration was investigated. The release of quercetin and berberine was determined by a Halo DB-20 spectrophotometer (Dynamica Scientific Ltd., Newport Pagnell, UK) at λ = 375 nm in the case of quercetin and λ = 340 nm for berberine. The concentrations of realized active compounds were calculated based on calibration curves.

### 2.6. Fruit Storage and Firmness Method

**Figure 2:**
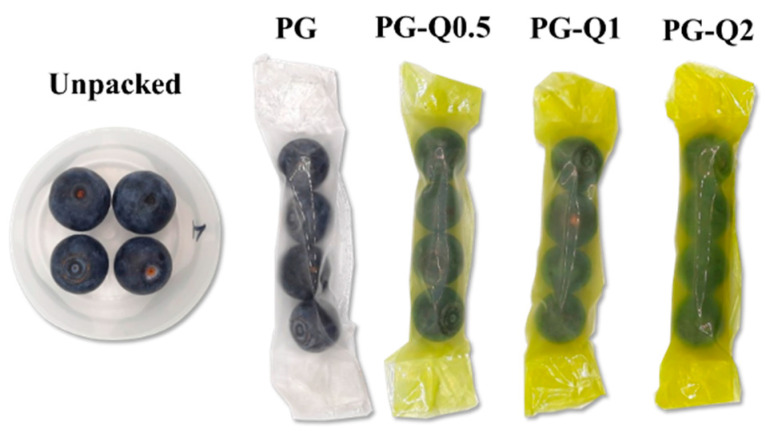
Photographs of the unpacked (reference) blueberries and blueberries packed into the prepared polymeric films.

Fresh blueberries (Chile origin, Brightwell variety), with no visible physical damage, were selected and packed into the studied polymeric films: PG and PG incorporated with quercetin or berberine. Two packs for each formulation were prepared, and each pack contained 4 blueberries (Figure 2). Formed packages were stored for 7 days in the same condition as samples during WVTR measurements. A gravimetric measurement of the packages was performed every 24 h to test the weight loss, and the firmness of the blueberries was tested before and after 7 days of storage. The mechanical tests were conducted using an EZ-SX texture analyzer (Shimadzu, Kyoto, Japan), equipped with a 50 N load cell. A cylindrical probe (diameter 20 mm) was used to investigate the behavior of blueberries under squeezing. The squeezing response was tested, recording the force necessary to obtain 20% compression of the fruits.

### 2.7. Analysis of UV Protection

Samples in the form of rectangles (1 × 3 cm) were used to observe the optical properties of the PG sample as well as PG-quercetin and PG-berberine films. The spectra were collected within the range of 200–800 nm by using a Halo DB-20 (Dynamica Scientific Ltd., Newport Pagnell, UK) spectrophotometer operating in the absorbance mode. The transparency of films is reported as transmittance values at 600 nm. The UV-blocking activity was evaluated in the *UV−A* and *UV−B* ranges by using the following Equations (3) and (4):

(3) $$UV-A_{blocking}=100-T_{UV-A}$$

(4) $$UV-B_{blocking}=100-T_{UV-B}$$

where *T_UV_−_A_* is the average transmittance value in the *UV−A* range (from 400 nm to 315 nm), and *T_UV_−_B_* is the average transmittance value in the *UV−B* range (from 315 to 280 nm).

## 3. Results and Discussion

### 3.1. Comparison of Antioxidative Activity

**Figure 3:**
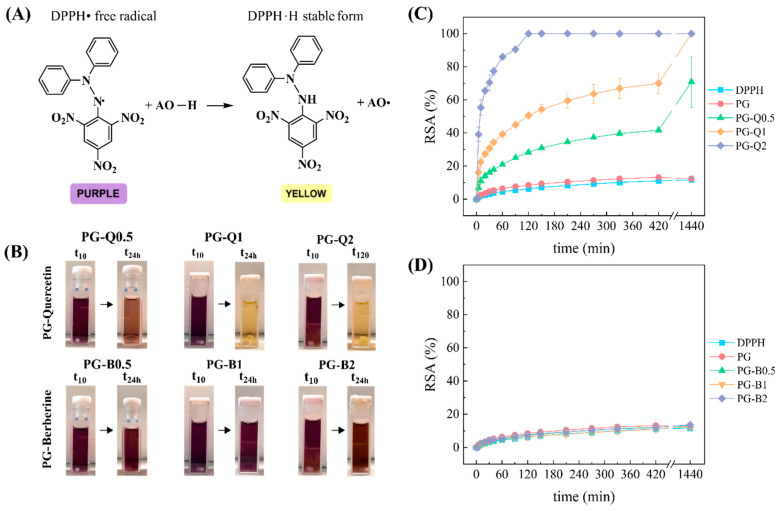
(**A**) DPPH scavenging reaction with the antioxidant. (**B**) Color transitions of the DPPH solution with PG-quercetin films compared to the PG-berberine films at different times of analysis. Radical scavenging activity (%) over time of (**C**) PG-quercetin films and (**D**) PG-berberine films.

Active packaging materials are expected to combine the property of passively protecting the food with more advanced characteristics with the aim of extending the food’s shelf life [17]. The antioxidative potential is one of the most important, as it suppresses the oxidation process by drastically reducing the concentration of free radicals in food [18,19]. Herein, the radical scavenging activity (RSA) of both PG-berberine and PG-quercetin films was assessed by using the DPPH• free radical scavenging method. Ethanol, which represents the food systems with the highest fat content [20], such as meat, was employed in order to enable the analysis. The aforementioned scavenging reaction is based on an electron transfer mechanism (ET), where the antioxidant reduces the DPPH• radical to its stable DPPH-H form (Figure 3A), leading to the bleaching of the absorption peak of the DPPH• radical: at about 517 nm. Figure 3C,D compare the response to the antioxidative potential of PG-berberine and PG-quercetin films. It is worth noting that the two additives are characterized by a completely different behavior, as strong antioxidative activity is exhibited by PG-quercetin films, while PG-berberine films display a much less significant potential. This may be linked to the different chemical structures of the two molecules. Quercetin belongs to the category of flavonoids, which are well-known in the literature as good antioxidants [21], related to typical hydroxyl groups present in a catechol or pyrogallol structure [22]. On the other hand, berberine is a natural isoquinoline alkaloid. Although evidence has shown it has excellent oxidative stress-reducing properties [23,24], its oxidation-reducing efficacy as a free scavenger is insufficient when compared to its derivatives, which contain phenolic hydroxyl groups [25].

The visible differences in the antioxidative characteristics of PLA-based films containing different flavonoids can also be affected by different solubility of both berberine and quercetin, in EtOH, a solvent used in the DPPH method in this study. The previous research on the characteristics of both quercetin and berberine showed relatively low mole fraction solubility in pure ethanol, equal to 135.00 × 10^−5^ at 298.6 K for quercetin [26], and 0.444 × 10^−3^ at 298 K in the case of berberine [27]. However, LanQing Li et al. [28], who applied EtOH as a solvent in the extraction of both flavonoids from the soil, found the highest affinity of quercetin toward ethanol.

Quercetin showed its ability to scavenge the free radicals in a concentration-dependent manner, as Figure 3B reveals. Even if PG-Q0.5 never reaches 100% of RSA, it scavenges 70% of the DPPH in 24 h. Doubling the amount of additive, 24 h was enough to reach the plateau, and the RSA of 70% was attained after 7 h of analysis. Finally, PG-Q2 exhibited the strongest antioxidative capacity, ensuring the total color transition from purple to yellow within the first 2 h of analysis (Figure 3B).

According to the obtained results, PG-quercetin films revealed a powerful oxidation-impeding activity in ethanol, which can, in turn, hinder the spoilage of food ingredients that would otherwise be exposed to oxygen, therefore resulting in the extension of the shelf life of food products.

### 3.2. Resistance to Water Vapor Permeation

**Figure 4:**
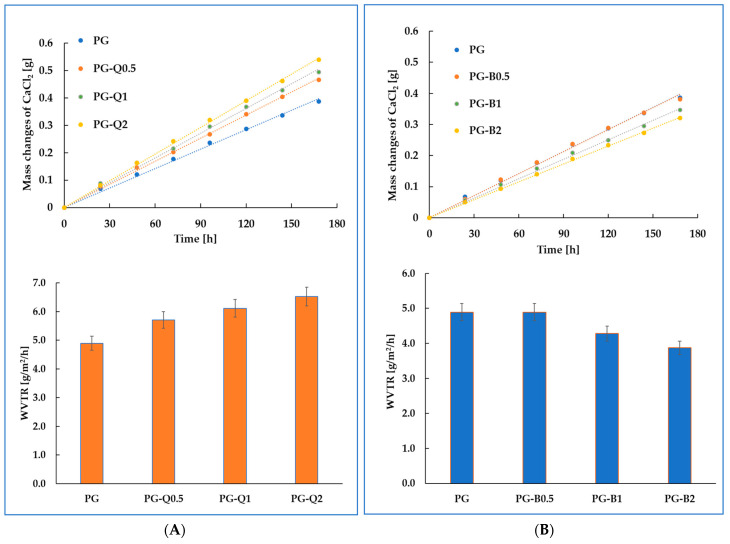
Changes in the mass of CaCl_2_ and WVTR materials containing (**A**) quercetin and (**B**) berberine.

Water vapor permeability is one of the fundamental parameters taken into account when choosing food storage packaging. It may determine the quality and possible storage time of products. Water vapor permeability is influenced by the type of polymer and crystal-amorphous structure, as well as the type and hydrophilic-lipophilic nature of the introduced additives [29]. In the case of the obtained materials, it could be observed that the type of introduced flavonoid determined the water vapor permeability of the formed PLA-based films. Figure 4A,B show changes in the mass of calcium chloride during the analysis and WVTR values for the tested films. The introduction of berberine in an amount of 1–2% wt. reduced the WVTR value. This was related to the formation of branched berberine structures on the surface of the films (Figure S1), as well as an increase in the degree of crystallinity of the obtained materials, which was observed and described in our previous work [12]. It was established that the introduction of 2% wt. berberine changed the degree of crystallinity from 1.8% to 10.7%. On the contrary, the incorporation of quercetin into the PLA-PEG system leads to an increase in water vapor permeability. Previous research has shown that the addition of quercetin slightly reduces the degree of crystallinity of the resulting packaging materials [11]. It was observed that the incorporation of 2% wt. of quercetin changes the degree of crystallinity by about 0.4%. The decrease in the crystallinity of the tested materials may very likely be the reason for the increased water vapor permeability of films containing quercetin. Interestingly, the increase in water vapor transmission rate was observed despite the hydrophobic nature of quercetin, which, when incorporated into chitosan, resulted in a slight decrease in the WVTR value [4]. Yadav et al. made similar observations in their work [30]. They investigated the physicochemical and biological characteristics of chitosan-gelatin-based film filled with quercetin, used as food packaging. However, the addition of quercetin to polyethylene did not affect water vapor permeability [31]. Based on the obtained results, it was indicated that the molecular structure of polymeric material was mainly responsible for the barrier properties of the composite.

Summarizing, the comparison of the properties of materials containing quercetin brought us to the conclusion that the WVTR values significantly depend on the type of applied polymer matrix.

### 3.3. Results for Specific Migration

**Figure 5:**
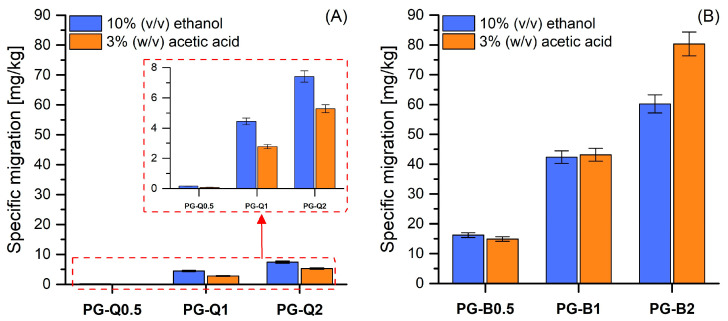
Migration of (**A**) quercetin and (**B**) berberine into food simulants (ethanol and acetic acid).

The introduction of active compounds into the polymer matrix involves the risk of releasing them into the package environment and then directly into the packaged product. Taking into account that the added compounds may constitute a threat not only to pathogenic bacteria but also to human health and life, migration limits have been established for various compounds contained in the packaging. Along with the quantity regulation, conditions for migration analysis were established depending on the type of packaged food and storage conditions. For this purpose, the compositions of food-simulating liquids were determined and assigned the letters A to D. Liquid A is 10% (*v*/*v*) ethanol, liquid B is acetic acid 3% (*w*/*v*), C 20% (*v*/*v*) ethanol, and D1 and D2 were 50% (*v*/*v*) ethanol and isooctane, respectively. Food simulants marked with the letters A, B, and C are intended for food that is hydrophilic [32]. Additionally, food simulant A is used for fruit that has not been peeled and sliced, while liquid B should be used for those foods that have a pH below 4.5. Due to the fact that in accordance with the regulation, liquids A and B are suggested to simulate fresh or chilled storage fruit, the migration of the used flavonoids was tested in these liquids in conditions that included storage at room temperature and at a reduced temperature. Figure 5 shows the results for the specific migration of quercetin and berberine in the studied food simulants. Based on the data presented in Figure 5A, it can be concluded that the migration of quercetin to both ethanol and acetic acid aqueous solutions meets the requirements set out by the European Union regulations because its migration in the case of storing food containing water or alcohol does not exceed 60 mg/kg of stored food. However, higher migration was observed with the type A liquid, i.e., liquid consisting of 10% (*v*/*v*) ethanol. The same analysis performed on materials containing berberine indicated that the assumed standards were significantly exceeded, even with small amounts of berberine (Figure 5B) introduced into the PLA-PEG system. The obtained results allow us to conclude that the addition of berberine in the amount of 0.5% wt. causes its migration at a level of 16–18 mg/kg into both tested liquids, while the introduction of berberine in the amount of 2% wt. causes its migration at a level of 60 and 80 mg/kg into food-simulating liquids A and B, respectively. It is, therefore, evident that berberine, despite its excellent antibacterial properties [12], should not be used for food products containing water or with a pH below 4.5. It is very likely that it may be used in packaging intended for storing consumables containing fats.

The release of quercetin from polymeric films has already been studied in the works of Luzi et al. [33] and He et al. [34], who tested poly(vinyl alcohol)-based materials. However, in the mentioned works, samples were immersed in a simulant of fatty foods. Zhang and Zhao [35] described a study on quercetin migration from low-density polyethylene films. The procedure was performed in ethanol and acetic acid solutions at 0, 15, and 30 °C. It was established that migration of the used flavonoid was significantly higher in the case of food simulants based on ethanol than on acetic acid. The observation described by Zhang and Zhao is consistent with our results. Moreover, it should be noted that López-de-Dicastillo et al. [36] proved that the migration of active agents significantly depends on the affinity between antioxidants and food simulants. For this reason, quercetin easily migrated into the ethanol solution. 

Since berberine is mainly studied in the context of drug delivery systems and dressing materials, its release is most often tested using other substances. In the work of Yin et al. [37], research was carried out into the release of berberine into PBS buffer with different pH values. Similar studies on the release of berberine into PBS at pH = 7.4 were described by Zhang et al. [38]. In both cases, it was confirmed that materials containing berberine exhibited sustained drug-release ability.

### 3.4. Differences in Parameters of Stored Blueberries

**Figure 6:**
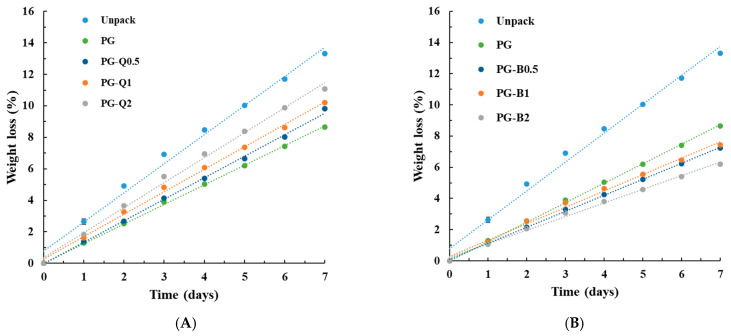
The mass changes of blueberries stored in packaging containing (**A**) quercetin or (**B**) berberine.

Compounds characterized by antibacterial and antioxidant properties are introduced into the polymer matrix to extend the firmness and freshness of stored food. They can protect fruits and vegetables against loss of nutritional properties and proliferation of bacteria and fungi. In this study, the blueberry, which is a healthy and tasty fruit, was selected for research. Additionally, it contains anthocyanins and natural plant compounds that have strong antioxidative properties and may have a beneficial effect on health. It should also be noted that blueberries are small, which is important during storage. The weight loss of blueberries stored in packaging made of films containing quercetin (Figure 6A) or berberine (Figure 6B) was evaluated.

**Figure 7:**
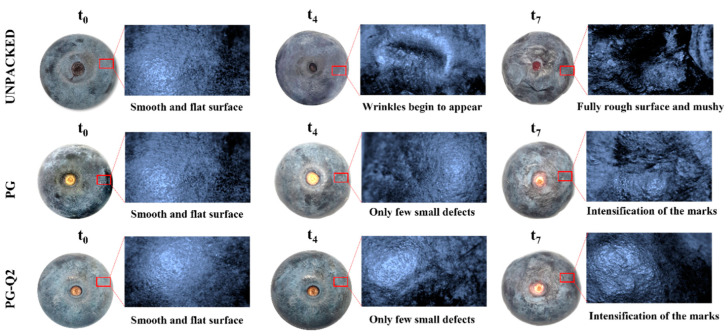
Examples of surface changes of blueberries during storage.

The obtained results indicate that the greatest weight loss was observed in blueberries without packaging. Particular active additives affect the storage of fruit but in very different ways. The introduction of quercetin into the PLA-PEG system slightly increased the weight loss of blueberries; however, the noted values were still lower than those of the unpackaged fruits. On the other hand, the addition of berberine significantly reduced the weight loss of the tested fruits. It is known that fruit loses a significant amount of water through respiration and transpiration. Packaging made of materials with increased resistance to water vapor transmission results in limited moisture loss of stored fruit. Taking into account the fact that the addition of berberine to the PLA-PEG system reduces WVTR, it is not surprising that in the case of blueberries stored in packaging made of materials containing berberine, moisture loss is reduced. The loss of moisture by blueberries directly determines the firmness of the fruit, which is clearly illustrated by the sample photos (Figure 7) of changes in the structure of the peel of unpacked blueberries, blueberries stored in packaging without an active additive and in a material containing 2% wt. quercetin. Firmness is a texture feature expressing the material’s resistance to deformation. It is one of the most easily measurable texture components, and therefore it is one of the most important indicators of fruit quality. It allows the determination of changes in fruit quality during storage and trade. Firmness may be an indicator of the fruit’s resistance to mechanical damage. It is also a useful parameter in the process of mathematical modeling and predicting quality changes required by the market [39]. It should be noted that the features that decide the compression results include the firmness of the pulp, the mechanical strength of the peel, the turgor pressure of the fruit, and the viscosity of the juice [40].

**Figure 8:**
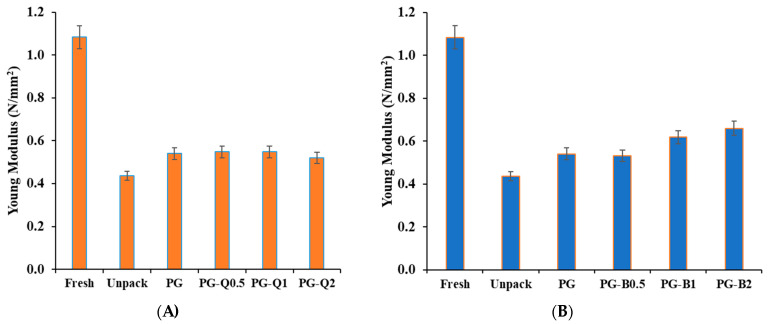
Changes in Young’s modulus of fruit stored in films containing (**A**) quercetin and (**B**) berberine.

Figure 8A,B present the results of the compression test performed on blueberries. Young’s modulus was used as an indicator of firmness. The obtained results confirmed that the packaging provided significant protection for extending the firmness of the fruit. In the case of Young’s modulus values for blueberries stored in packaging made of films containing berberine, greater firmness was observed compared to fruit packed in pure PG film. 

This is presumably due to the reduced water vapor permeability of packaging with the addition of berberine, which translates into lower weight loss and higher Young’s modulus values. However, in the case of fruit packed in films with the addition of quercetin, although the addition of quercetin increased water vapor permeability and the weight loss was greater in the case of blueberries stored in packaging containing quercetin, the firmness of the fruit was comparable to that of fruit stored in PG packaging. This can be attributed to the fact that materials containing quercetin are characterized by significant antioxidant properties, which may help stored fruit remain fresh.

The process of storing blueberries has also been described by other scientists. Li et al. [41] analyzed the effect of packaging consisting of starch films reinforced with curcumin-loaded nanocomplexes on the weight loss, firmness, and appearance of blueberries. It was established that the applied packaging seemed to be a promising method of improving the nutritional and commercial value of stored fruit. Zhao et al. [42] described blueberry preservation by means of active materials based on PVA/Tremella polysaccharide and soy protein isolate complex/ε-polylysine. The weight loss, decay rate, as well as vitamin C content of blueberries were taken into account to evaluate the quality of stored fruits. In conclusion, it was indicated that the applied packaging could postpone senescence and effectively prolong the shelf life of blueberries. The application of Aloe vera polysaccharide-based packaging films in blueberry preservation was studied in the work of Tang et al. [43]. It was found that the used materials improved the resistance of the fruit against external forces. In the work of Mannozzi et al. [44], the effects of chitosan-based coatings enriched with procyanidin by-products on the quality of fresh blueberries during storage were analyzed. The blueberries’ quality was evaluated based on the changes in color, weight loss, dry matter, pH, firmness and antioxidative activity. The obtained results allowed for a positive assessment of the coatings used. Similar observations were made by the research described in this paper, which indicated that formed packaging containing quercetin or berberine protects blueberries and extends their shelf life.

### 3.5. UV Protection

**Figure 9:**
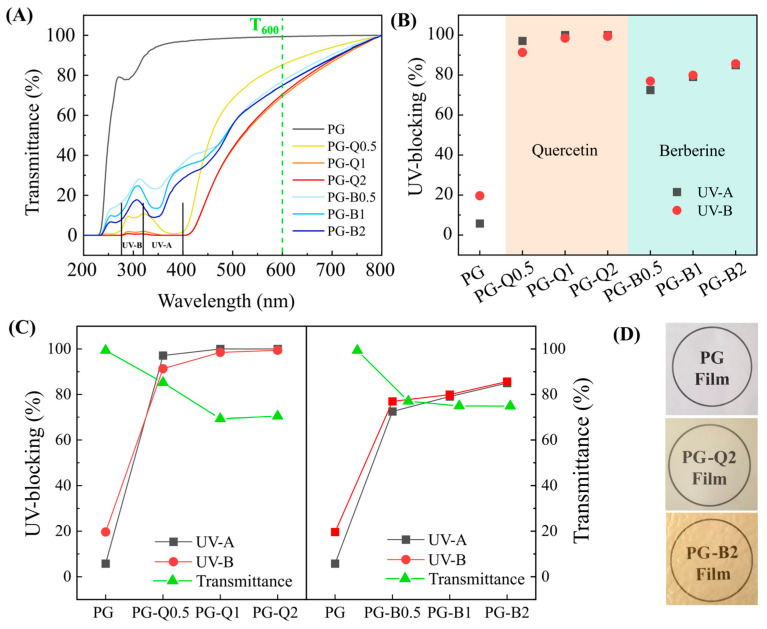
Optical properties of studied materials. (**A**) The UV-visible spectra of PG and PG-berberine and PG-quercetin samples. (**B**) UV-blocking factors of the samples in the UV-A (400–315 nm) and UV-B (315–280 nm) ranges. (**C**) Comparison of the transparency values with the UV-blocking factors of the samples in the UV-A (400–315 nm) and UV-B (315–280 nm) ranges. (**D**) Representative photos on a printed text of PG, PG-Q2 and PG-B2 samples.

In the case of packaging materials, UV protection is as important as transparency because it guarantees a longer shelf life for the packaged contents. It is well known that UV light causes photo-oxidative degradation, a reaction that can adversely affect the taste as well as the odor or color of food products. As a result, the shelf life of a given product is reduced. Moreover, if consumers have a negative experience with the product, the manufacturer’s reputation may also suffer. In Figure 9A, the UV−visible absorption spectra of the studied materials are depicted. It can be clearly seen that the incorporation of the discussed flavonoids significantly influences UV-blocking (Figure 9B).

The addition of berberine into the PLA-PEG system improved the protection the obtained films offered against UV radiation. However, the effect did not cover the whole range of UV-A and UV-B, reaching a maximum of 85% of protection for PG-B2. On the other hand, the presence of quercetin in the PG matrix allowed for almost complete protection, specifically of 97% against UV-A and 91% against UV-B after introducing only 0.5% wt. of the active compound, and reached 100% with 2% wt. in the whole UV range. The obtained protection can be assigned to the remarkable ability of phenolic groups responsible for blocking UV radiation [45,46]. The analyzed materials showed good transparency even at the highest concentrations tested (Figure 9D), and this parameter was discussed in more detail in our previous works [11,12]. Since providing a clear view of the packaged items while protecting them from UV radiation is essential for food packaging applications, Figure 9C reports the correlation between these two parameters as a function of the increasing content of quercetin and berberine. In general, It can be clearly seen that higher UV protection corresponds to lower transparency of the materials. However, increasing the flavonoid content, transparency seems to stabilize at 80% for berberine at 2% wt. and 70% for quercetin at 2% wt., while guaranteeing high levels of UV-blocking activity.

## 4. Conclusions

This study involved a comparison of the influence of two different flavonoids—quercetin and berberine—on the most important factors affecting food storage. The recorded data clearly indicated that packaging containing quercetin presents significantly higher antioxidative properties than films with the addition of berberine. On the other hand, materials containing berberine are characterized by an improvement in resistance to water vapor transmission compared to films incorporating quercetin. It should be stressed that the introduction of berberine and quercetin substantially delays the decline of blueberry quality and constitutes protection against UV radiation. Additionally, taking into account that migration of quercetin into food simulants (3% *w*/*v* acetic acid and10% *v*/*v* ethanol) is below 10 mg/kg, it can be concluded that materials consisting of PLA-PEG and quercetin may be approved by the EU Commission for food storage.

All performed studies indicated that PLA-based films containing quercetin and berberine are promising materials, with quercetin extending the shelf life of fruit and vegetables and berberine preserving oil-based consumables.

## Figures and Tables

**Table 1:** Compositions of investigated materials (P—polylactide; G—PEG, Q—quercetin, B—berberine).

Sample	Flavonoid Content % wt. *	PEG Content % wt. *
PG	-	5
PG-Q0.5	0.5	5
PG-Q1	1	5
PG-Q2	2	5
PG-B0.5	0.5	5
PG-B1	1	5
PG-B2	2	5

* the percentage of additive in relation to PLA mass.

## Data Availability

Data is contained within the article or supplementary material.

## Supplementary Materials

The following supporting information can be downloaded at https://www.mdpi.com/article/10.3390/polym16111577/s1, Figure S1: Morphology of the films with the addition of berberine.
