# Supplementary figures and images for: Comparison of Polylactide-Based Active Films Containing Berberine and Quercetin as Systems for Maintaining the Quality and Safety of Blueberries
Source: Polymers (Basel). 2024 Jun 2;16(11):1577. doi: 10.3390/polym16111577 (PMC11174692; doi:10.3390/polym16111577)

Supplementary materials

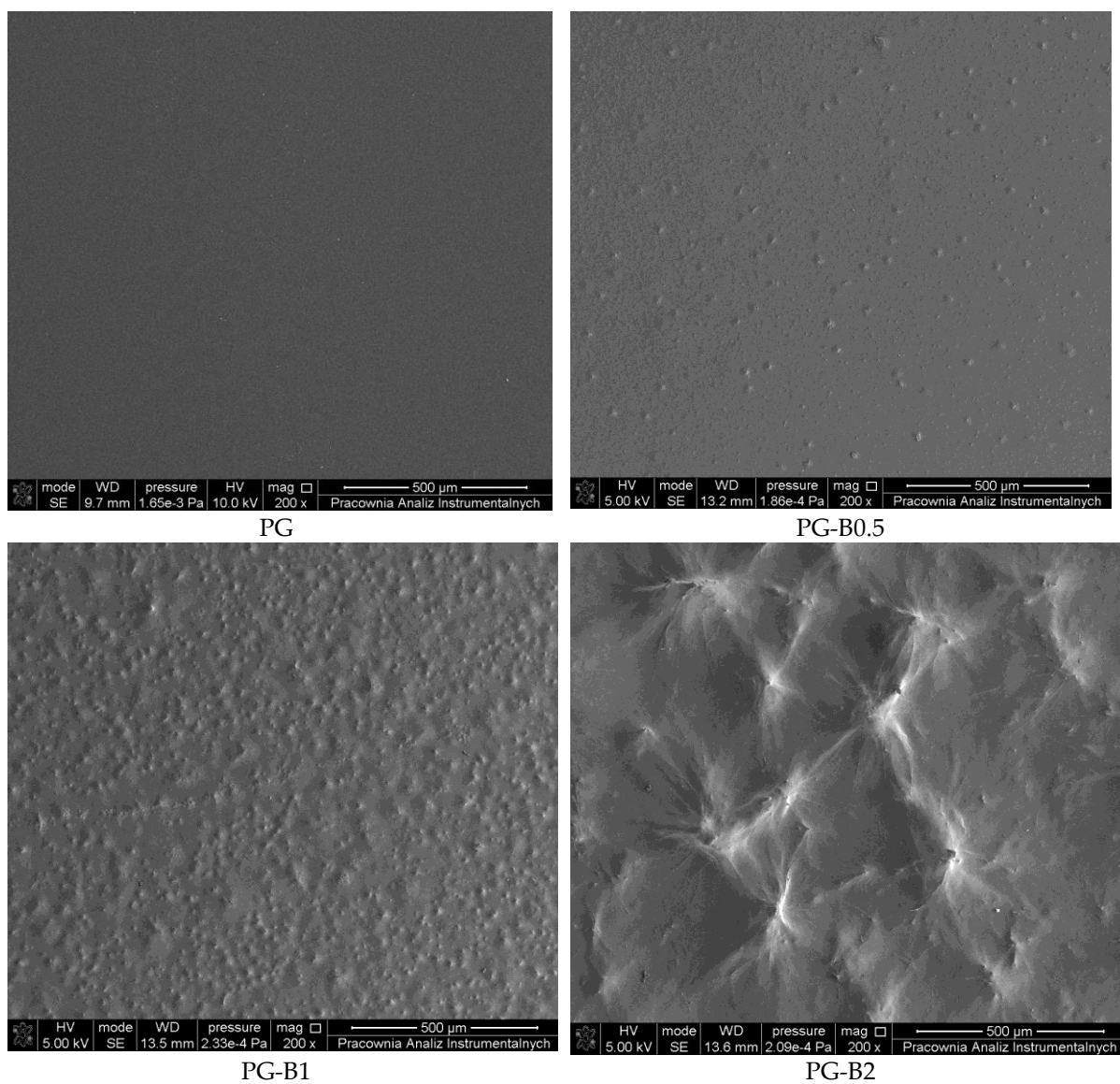

**Figure S1.** Morphology of the films with an addition of berberine.

Supplement: Supplementary file 1 [file polymers-16-01577-s001.zip › polymers-3019217-supplementary.pdf]
